# Cut-Off Value of Thyrotropin-Receptor Antibodies in Grave’s Disease in Basrah

**DOI:** 10.7759/cureus.47708

**Published:** 2023-10-26

**Authors:** Maher A Radhi, Nassar T Alibrahim, Abbas A Mansour

**Affiliations:** 1 Diabetes and Endocrinology, Faiha Specialized Diabetes, Endocrine and Metabolism Center, Basrah, IRQ; 2 Diabetes and Endocrinology, University of Basrah, College of Medicine, Basrah, IRQ

**Keywords:** iraq, thyroid, cut-off, thyrotropin receptor antibodies, grave’s disease

## Abstract

Background: The diagnosis of Grave's disease (GD) poses a challenge. Thyrotropin-receptor antibodies (TRAb) are the key diagnostic feature of GD, as the American and European Thyroid Associations suggested.

Aim of the study: This study aims to find a cut-off level of TRAb in GD in Basrah.

Methods: This is a retrospective study that included 617 patients with hyperthyroidism (530 GD and 87 non-Grave's disease (NGD) (thyroiditis or subclinical hyperthyroidism)). The candidates were patients presenting with hyperthyroidism who were referred for TRAb assay, while patients with thyroid carcinoma or nodular thyroid disease, pregnant ladies, and patients who were treated were excluded.

Results: The manufacturer cut-off value of 1.75 IU/L had a sensitivity of 88.1%, specificity of 72.4%, positive predictive value (PPV) of 95.1%, and negative predictive value (NPV) of 50.0%. Our data analysis through receiver operating characteristic (ROC) statistics revealed that the optimum cut-off point with the highest total sensitivity and specificity was determined to be 3.95 IU/L, as it had a sensitivity of 76.9%, specificity of 98.8%, PPV of 99.7%, NPV of 41.3%.

Conclusion: For a more accurate diagnosis of GD, the findings of the present study support the implementation of a higher TRAb cut-off value (3.95 IU/L) than that predefined by the manufacturer (1.75 IU/L).

## Introduction

Graves' disease (GD), an autoimmune disorder, is widely recognized as the predominant etiology of hyperthyroidism in regions with adequate iodine levels [1]. Additionally, it is linked to dermopathy and orbitopathy. The etiology of the condition may be attributed to the presence of thyrotropin-receptor antibodies (TRAb), which elicit a stimulatory effect on the thyroid-stimulating hormone (TSH) receptor located on the outer membrane of thyroid follicular cells. Therefore, TRAb plays a central role in developing GD [2]. Thus, the presence of TRAb in the majority of individuals with GD underscores its significance in distinguishing GD from other etiologies of hyperthyroidism.

The TRAb are mostly produced by B lymphocytes located inside the thyroid cells. Additionally, they can be synthesized inside lymph nodes and bone marrow. B lymphocytes are activated by T cells that become sensitized by antigens present in the thyroid gland. The thyroid-stimulating immunoglobulins interact with the TSH receptor located on the cell membrane of the thyroid gland, therefore initiating the activation of the TSH. It elicits both the manufacturing of thyroid hormones and the growth of the thyroid gland, resulting in the development of hyperthyroidism and thyromegaly [3].

Additional etiologies of hyperthyroidism often seen in clinical settings include different types of thyroiditis, autonomously functioning thyroid nodules, toxic multi-nodular goiter, gestational thyrotoxicosis, and exogenous administration of thyroxine. In regions with adequate iodine levels, the primary etiology of hyperthyroidism is GD, which is subsequently followed by nodular thyroid disease and thyroiditis [4].

The differentiation among different etiologies of hyperthyroidism has significance due to the varying therapeutic approaches associated with each cause. The differential diagnosis of hyperthyroidism is conducted by using a comprehensive approach that includes gathering patient history, doing a thorough clinical examination, performing biochemical investigations, utilizing thyroid scintigraphy, administering the TRAb test, conducting thyroid ultrasonography with Doppler study, and closely monitoring the patient's progression throughout the natural course of the condition [5]. The American and European Thyroid Associations both suggest the use of TRAb in the diagnostic process of GD [6,7].

Nevertheless, there are some constraints in the interpretation of TRAb. This encompasses the many test methodologies used, the bioactivity characterization of TRAb, and the detection and prevalence of TRAb in individuals afflicted with other autoimmune disorders, thyroid-related ailments, and even non-autoimmune conditions. In addition, it should be noted that the cut-off values of TRAb for achieving optimal diagnostic accuracy might differ according to the individual test type and manufacturer. Consequently, this variability in cut-off values can result in varying levels of sensitivity and specificity for the assays. The diagnosis of GD might significantly impact the care of individuals with reduced TSH levels in relevant subjects [8].

## Materials and methods

Study design

This retrospective study was conducted at the Faiha Specialized Diabetes, Endocrine, and Metabolism Center (FDEMC). Data were collected from the database registry of patients who had attended the FDEMC for hyperthyroidism during the period from 2013 to 2023.

Inclusion criteria

The inclusion criteria were patients presenting with hyperthyroidism who were referred for TRAb assay by their treating endocrinologist and were later classified as GD (530 cases) and 87 with NGD (thyroiditis or subclinical hyperthyroidism).

Exclusion criteria

Patients with the following conditions were excluded: thyroid carcinoma, nodular thyroid disease, pregnant ladies, patients treated before being sent for TRAb assessment, and patients with incomplete data registry.

Selection of research population

Convenient sampling was employed for this study, as the selected population were patients who satisfied the inclusion criteria during the study period. Among 669 cases, 617 cases were selected, while 52 were excluded due to the reasons mentioned in the exclusion criteria.

Diagnosis of GD was in concordance with the guidelines of the American Thyroid Association, which require the presence of hyperthyroidism and one or more of the following features: (1) detectable TRAb in the serum, (2) evidence of ophthalmopathy and/or dermopathy; and (3) increased radioactive iodine uptake or ultrasound features consistent with GD [7].

Ultrasound features suggestive of GD were an enlarged hyperechoic gland, thyroid echotexture exhibiting heterogeneity, hypervascularity, and the relative absence of nodularity [9].

The diagnosis of thyroid eye disease was established based on one of the following two criteria [10]: (1) When eyelid retraction is present, the diagnosis is determined by the additional presence of abnormal thyroid function, exophthalmos, optic nerve dysfunction, or involvement of the extraocular muscles. Other potential causes must be ruled out to provide an accurate diagnosis. (2) If there is no eyelid retraction, the diagnosis is determined by the occurrence of exophthalmos, optic nerve dysfunction, or extraocular muscle involvement in the context of abnormal thyroid function.

Laboratory measurement

The TRAb test was carried out using the chemiluminescent immunoassay (Elecsys Anti-TSHR, Roche Diagnostics, Rotkreuz, Switzerland) kit on the Cobas e411 analyzer series (Roche Diagnostics, Rotkreuz, Switzerland).

Statistical analysis

Data was summarized using measures of frequency (mean) and dispersion (standard deviation), tables, and graphs. Analysis was performed using SPSS Statistics version 26 (IBM Corp. Released 2019. IBM SPSS Statistics for Windows, Version 26.0. Armonk, NY: IBM Corp.). Receiver operating characteristic (ROC) curve analysis was used to determine the optimum cut-off value (cut-off value with the highest total of sensitivity and specificity).

## Results

A total number of 617 participants were included (530 GD and 87 NGD). Basic characteristics are illustrated in Table 1. Patients with GD had significantly lower BMI and TSH and higher TRAb, T3, and FT4 levels than those with NGD.

**Table 1:** Basic sociodemographic and clinical characteristics of GD and NGD BMI: body mass index, TRAb: thyrotropin-receptor antibodies, FT4: free thyroxine, T3: triiodothyronine, TSH: thyroid-stimulating hormone, SD: standard deviation, *Mann–Whitney U test, **Fisher’s-exact test

Basic characteristics	Groups		p-value
	GD (N=530)	NGD (N=87)	
Age			
Mean ± SD	37.2 ± 13.5	38.22 ± 13.5	0.645
Sex			
Males	180 (34.0%)	15 (17.2%)	0.002
Females	350 (66.0%)	72 (82.8%)	
BMI			
Mean ± SD	26.1 ± 6.3	29.1 ± 9.9	<0.001
TRAb			
Mean ± SD	13.5 ± 12.0	1.3 ± 1.1	<0.001
TED			
Yes	71 (13.4%)	0 (0.0%)	<0.001
No	459 (86.6%)	86 (86.0%)	
FT4			
Mean ± SD	3.8 ± 2.1	1.27 ± 0.22	<0.001
T3			
Mean ± SD	3.2 ± 1.7	2.5 ± 1.6	<0.001
TSH			
Mean ± SD	0.009 ± 0.1	0.021 ± 0.02	<0.001

A total number of 87 (64 thyroiditis and 23 subclinical hyperthyroidism) NGD participants were included, as shown in Table 2.

**Table 2:** Diagnosis of patients with NGD

Diagnosis	Frequency	Percentage
Thyroiditis	64	73.6%
Subclinical hyperthyroidism	23	26.4%
Total	87	100.0%

**Figure 1:**
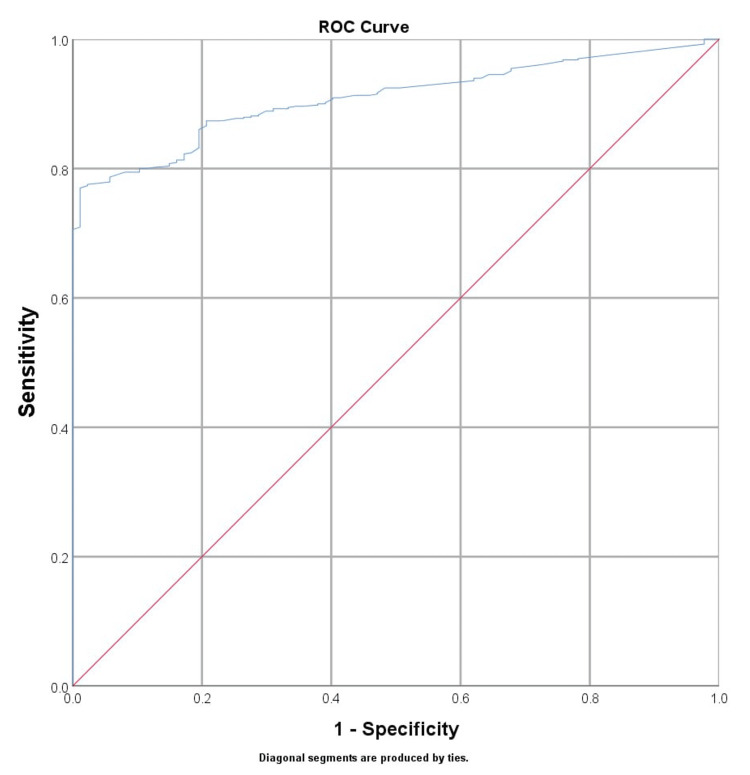
ROC analysis diagnostic indices of TRAb for prediction of GD ROC: receiver operating characteristic, TRAb: thyrotropin-receptor antibodies

To evaluate the optimum cut-off value for TRAb, an ROC analysis was employed (AUC = 0.927, p-value <0.001). The optimum cut-off point with the highest total sensitivity and specificity was determined to be 3.95 IU/L, as shown in Figure 1 and Table 3.

**Table 3:** Sensitivity, specificity, PPV, and NPV of TRAb at cut-off ≥1.75 IU/L and ≥3.95 IU/L PPV: positive predictive value, NPV: negative predictive value, TRAb: thyrotropin-receptor antibodies

Statistic	≥1.75 IU/L	≥3.95 IU/L
Sensitivity	88.1%	76.9%
Specificity	72.4%	98.8%
PPV	95.1%	99.7%
NPV	50.0%	41.3%

The optimum cut-off point with the highest total of sensitivity and specificity was determined to be 3.95 IU/L as it had a sensitivity of 76.9%, specificity of 98.8%, positive predictive value (PPV) of 99.7%, and negative predictive value (NPV) of 41.3%. On the other hand, the manufacturer cut-off value of 1.75 IU/L had a sensitivity of 88.1%, specificity of 72.4%, PPV of 95.1%, and NPV of 50.0%, as shown in Tables 4-5.

**Table 4:** Accuracy of a 3.95 IU/L cut-off point of TRAb in the differentiation between GD and NGD TRAb: thyrotropin-receptor antibodies, GD: Grave's disease, NGD: non-Grave's disease

TRAb	Groups		Total
	GD	NGD	
≥3.95 IU/L	408	1	409
<3.95 IU/L	122	86	208
Total	530	87	617

**Table 5:** Accuracy of the standard 1.75 IU/L cut-off point of TRAb in the differentiation between GD and NGD TRAb: thyrotropin-receptor antibodies, GD: Grave's disease, NGD: non-Grave's disease

TRAb	Groups		Total
	GD	NGD	
≥1.75 IU/L	467	24	491
<1.75 IU/L	63	63	126
Total	530	87	617

## Discussion

The diagnosis of GD encompasses two distinct dimensions of complexity. The lack of standardized guidelines for diagnosing GD is a notable concern. Furthermore, the diagnosis of GD cannot completely rely on clinical symptoms and imaging techniques since no distinct characteristic may differentiate GD from other forms of hyperthyroidism. For instance, the presence of diffuse uptake on a scintigraphy scan corresponds to the diagnosis of GD; however, it is important to note that this characteristic might also resemble the manifestation of recovering thyroiditis [11]. Hence, the diagnosis is ultimately determined by the clinician's comprehensive assessment, which may include clinical follow-up.

The challenge in distinguishing GD from various etiologies of hyperthyroidism highlights the need to do TRAb assessments. In addition, this underscores the significance of using accurate threshold values for TRAb tests, given that the majority of clinical diagnoses rely on the results obtained from this particular assay.

In the present study, the optimum cut-off point (3.95 IU/L) had a sensitivity of 76.9% and specificity of 98.8%. The study by Smit et al. calculated that a cut-off of 4.5 IU/L had a sensitivity of 96.4% and specificity of 90.9% [11]. Xu et al. reported an optimum cut-off that had 1.245 IU/L had a sensitivity of 96.6% and specificity of 97.1% [12]. Kamijo et al. determined that a cut-off value of 1.5 IU/L had a sensitivity of 96.2% and specificity of 95.2% in the differentiation between GD and painless thyroiditis [13]. John et al. revealed that a cut-off of 3.37 IU/L had a sensitivity of 90.2% and specificity of 90.1% [8].

The findings of our study provide evidence in favor of implementing a higher threshold for TRAb in clinical laboratory settings. This preference is driven by our prioritization of achieving a greater level of specificity. In the context of low-prevalence disorders, such as autoimmune diseases, a little decrease in specificity might result in an increased number of false positive results, whereas a slight decrease in sensitivity mostly leads to false negative results. Furthermore, the TRAb test mainly functions as a tool for confirmation rather than screening within the diagnostic framework for GD. It focuses on a parameter sometimes seen as pathognomonic when testing for the condition [14,15].

One study limitation is that thyroid scintigraphy was not used to diagnose GD because it was unavailable.

## Conclusions

For higher accuracy regarding the diagnosis of GD, the findings of the present study support the implementation of a higher TRAb cut-off value (3.95 IU/L) than that of the manufacturer cut-off (1.75 IU/L). This is because the concluded cut-off has demonstrated greater benefit in eliminating false positive cases. However, it is crucial to emphasize that when evaluating patients with probable GD, the TRAb test does not disregard the significance of the clinical context.
